# Long-acting reversible contraception and medication abortion: a national descriptive survey of Australian community pharmacist knowledge, attitudes and practices

**DOI:** 10.1007/s11096-026-02088-1

**Published:** 2026-02-24

**Authors:** Anisa Rojanapenkul Assifi, Sharon James, Satish Melwani, Kirsten I. Black, Angela Taft, Deborah Bateson, Wendy V. Norman, Danielle Mazza

**Affiliations:** 1 SPHERE, NHMRC Centre of Research Excellence, Monash University, Melbourne, Australia; 2 Department of General Practice, School of Public Health and Preventive Medicine, Monash University, Melbourne, Australia; 3 Specialty of Obstetrics, Gynaecology and Neonatology, Faculty of Medicine and Health, University of Sydney, Sydney, Australia; 4 Judith Lumley Centre, School of Nursing and Midwifery, Latrobe University, Melbourne, Australia; 5 Department of Family Practice, University of British Columbia, Vancouver, Canada; 6 Faculty of Public Health and Policy, London School of Hygiene and Tropical Medicine, London, UK

**Keywords:** Abortion, Community pharmacy, Contraception, Primary care, Women’s health

## Abstract

**Introduction:**

Community pharmacists can play a key role in the prevention and management of unintended pregnancy, including in the provision of counselling and by dispensing contraception, and for medication abortion (MA). However, Australian pharmacists’ practice and knowledge of effective contraceptive methods, including long-acting reversible contraception (LARC), is unknown, and few were registered to dispense MA at the time of the study.

**Aim:**

Our aim was to understand the knowledge, attitudes and practices of Australian community pharmacists in LARC and MA care.

**Method:**

We conducted a cross-sectional national online survey of community pharmacists from July until October 2021. Participants were recruited through convenience sampling via mail and partner organisations’ emails, newsletters, and mailing lists. We used descriptive statistical analysis, including counts, proportions, Pearson’s chi-squared tests and Poisson regression for data analysis. Our descriptive survey forms part of the Australian Contraception and Abortion Primary Care Practitioner Support Network (AusCAPPS) mixed-methods project (ACTRN12622000655741).

**Results:**

There were 533 eligible responses; 72% (n = 385) self-identified as women, and 71% (n = 378) were from metropolitan areas. Respondents' correct LARC knowledge varied, with 88% understanding LARC effectiveness, 67.7% understanding return to fertility, and 65.9% understanding LARC suitability for nulliparous women. Most pharmacists were registered to dispense MA (70%; n = 373), although fewer than half discussed LARC at the time of dispensing MA. Those working outside metropolitan areas were more likely to be registered to dispense MA and feel that they had the knowledge and confidence to dispense MA.

**Conclusion:**

With community pharmacists increasing scope of service in relation to contraception and MA, ongoing education and support will ensure they have accurate information for the provision of LARC and MA.

**Supplementary Information:**

The online version contains supplementary material available at 10.1007/s11096-026-02088-1.

## Impact Statements

- Pharmacists should be supported to include long-acting reversible contraception counselling during medication abortion dispensing, which, with uptake, can reduce repeat unintended pregnancies and improve patient outcomes.
- Pharmacists in rural and remote areas are taking steps to bridge critical gaps in abortion and contraceptive access. Further government support to strengthen their role will ensure equitable reproductive healthcare where it is needed most.
- To address knowledge gaps seen among younger pharmacists and to enhance culturally responsive care, additional training is needed within pharmacy undergraduate curricula to equip the profession to deliver inclusive, evidence-based care.

## Introduction

Universal access to sexual and reproductive health services is a key international priority, as outlined in the United Nations Sustainable Development Goal 3.7 [1]. This includes ensuring timely access to essential sexual and reproductive health services that support women in preventing unintended pregnancy, which is often associated with adverse physical and mental health outcomes for women and their children [2]. To prevent and manage unintended pregnancy, timely access to effective contraceptive methods such as long-acting reversible contraception (LARC) and abortion services is crucial. LARC methods, such as intrauterine devices (IUDs) and contraceptive implants, are the most effective contraceptive methods because of their high-efficacy, low user-dependence, and long duration [3, 4]. Additionally, medication abortion (MA) is a non-invasive alternative to surgical abortion to manage an unintended pregnancy. Post-MA access to a full range of contraceptive methods, including LARC methods, reduces the likelihood of repeat unintended pregnancy. The International Federation of Gynaecology and Obstetrics (FIGO) and The Royal College of Obstetricians and Gynaecologists (RCOG) similarly recommend that contraceptive services be offered in every abortion and post-abortion setting, and made available when the patient is ready to discuss contraception [5, 6].

Globally, 48% of pregnancies are unintended, with 61% of unintended pregnancies ending in abortion [7]. Additionally, delays in post-abortion contraception increase the risk of subsequent unintended pregnancy [8–12]. The World Health Organization highlights pharmacists’ role in improving women’s access to essential sexual and reproductive health services [13]. The expanding role of community pharmacists [14], has enabled the provision of bridging contraception to women [15], and the prescription of contraceptive methods [14, 16–19]. Research has shown that expanding pharmacists’ role in providing sexual and reproductive health services can significantly enhance women’s access to and continued use of effective contraceptive methods [15, 16, 20, 21].

In Australia, 40% of women experience an unintended pregnancy, with 31% of these resulting in abortion [22]. Australia’s National Women’s Health Strategy 2020–2030 identified prevention of unintended pregnancy as a key public health priority [23]. However, LARC uptake in Australia remains low (11%) compared to less effective contraceptive methods such as oral contraceptives (33%) and condoms (30%) [24]. Community pharmacists working in primary care settings, can dispense LARC and play an important role in supporting timely insertion by delivering person-centred effectiveness-based contraceptive counselling on LARC methods and ensuring product availability [25, 26].

Since 2003 in Australia, a mifepristone and misoprostol composite pack, called MS-2 Step, has been approved for use as MA up to nine weeks’ gestation [27]. MA up to nine weeks, also known as early medical abortion, is primarily provided and prescribed within primary healthcare services (e.g. general practice, family planning clinics) and dispensed by community pharmacists. Although MA is a safe and effective [13], until August 2023, pharmacists in Australia were required to individually register and undergo re-registration every three years to dispense this medication [28]. In 2021, only 13% of registered pharmacists were active dispensers of MA medicines [29, 30]. In 2020, telehealth was temporarily subsidised under the Australian government’s Medicare Benefits Scheme, allowing MA to be prescribed by general practitioners (GPs) at minimal cost. Their permanent continuation from July 1, 2023, underscores the need for community pharmacists to dispense MA medications, even when there are no local prescribers.

Community pharmacists are Australia’s most accessible health professionals, offering not only medication dispensing but also professional health services and advice [31]. In 2024, Australia had over 5900 community pharmacies, with 74% of regional/rural residents living within 2.5km of a pharmacy [32]. Community pharmacists are more accessible and equitably distributed than GPs [33]. Expanding LARC and MA services through pharmacists could improve the equitable prevention and management of unintended pregnancy. A 2020 survey of Australian community pharmacists examined contraceptive counselling in pharmacy practice. Pharmacists recognized contraceptive counselling as essential to their professional role, and while confident in advising about oral contraceptive pills OCP, they reported lower self-rated knowledge and confidence in other contraceptive methods. Additionally, the survey found that one-third of pharmacist respondents were approached weekly for contraceptive information [34]. Research on LARC and MA care provision by community pharmacists remains limited nationally and internationally. Given that individuals should have access to the full range of contraceptive methods and genuine choice, there is a need to better understand pharmacists’ knowledge, attitudes, and practices regarding the more effective LARC methods and MA. This study builds on the findings of the 2020 national survey [34] to address these gaps.

### Aim

Our aim was to understand the knowledge, attitudes and practices of Australian community pharmacists in LARC and MA care.

## Method

This cross-sectional online descriptive survey was conducted as part of the AusCAPPS (Australian Contraception and Abortion Primary Care Practitioners) Network, a national multidisciplinary online community of practice aimed at improving community pharmacists, practice nurses and GPs/family physicians provision of LARC and MA in primary care (ACTRN12622000655741) [35]. The Checklist for Reporting Results of Internet E-Surveys (CHERRIES) guided the reporting this study [36].

### Survey design

Our cross-sectional online descriptive survey was informed by previous LARC and MA surveys [37–39] and expert input from the trial’s investigators, pharmacy academics and sexual and reproductive health clinicians. The survey specifically focused on LARC and MA, as it builds upon the findings of a 2020 national survey focused broadly on contraception KAP and counselling [34]. The survey was further informed and piloted by community pharmacists, pharmacy students, and women’s health and primary care expert clinicians and academics.

The 50-question English questionnaire took approximately 10–15 min to complete (Supplementary 1). Demographic information (age, identifying gender, postcode, highest qualification, and duration of industry experience) was collected. Mandatory questions (e.g., “Are you a registered dispenser of medical abortion medicines (MS-2 Step)?”) were included with branching logic used to ask adaptive questions depending on previous responses (e.g., clarifying the number of years of dispensing MS-2 Step if they were a registered dispenser). Responses included a mix of nominal and ordinal values, including 3- and 5-point Likert scales. For further elaboration, single-line free-text responses were invited if ‘other’ was selected to a question (e.g., “What factors influence you to recommend long-acting reversible contraceptives to an eligible patient?”). Respondents could review answers before submission.

### Study setting, sampling and recruitment

Australian Health Practitioner Regulation Agency verified community pharmacists working in Australia at the time of the survey were invited to participate. A target sample of 500 responses was sought, using convenience sampling, to represent key characteristics of Australian community pharmacists, and to reflect their knowledge, attitudes and practices with a precision of ± 5%.

Recruitment included email and mail-outs to database contacts (built from publicly available pharmacy contact details) and dissemination of recruitment materials through discipline-specific professional networks mailing lists, including the Pharmaceutical Society of Australia and family planning organisations. Further advertising was conducted through social media, including Facebook and Monash Pharmacy Alumni LinkedIn Group.

An explanatory statement was provided to respondents prior to accessing the survey. Consent was implied by survey completion. After completing the survey, respondents were invited to join the AusCAPPS Network.

Given the AUD$40 reimbursement offered to respondents, verification measures were implemented, including matching Australian Health Practitioner Regulation Agency registration numbers to email addresses and a variety of human- and computer-based anti-fraud strategies, including a honeypot question, reCAPTCHA and manual screening by researchers for illogical or duplicate data. Once validity was confirmed, respondents received their electronic voucher via email.

### Data collection and data analysis

The online survey ran from 19th July to 5th October 2021, when the desired sample size was reached. All survey responses were captured and stored by REDCap (Research Electronic Data Capture), an electronic data capture tool hosted and managed by Helix (Monash University) [40, 41]. Respondents' identifiable information was collected and stored separately from the survey data. All data were accessible only to the research team and stored on a password-protected university drive in accordance with university policy.

Survey data were exported from REDCap to Microsoft Excel for response cleaning and verification, then exported to STATA Statistical Software (Version 18) [42] for further management and analysis. Descriptive statistical analysis, including counts, proportions, Pearson’s Chi-Squared tests and Poisson regression, were undertaken. Participants’ location based on remoteness and population size was analysed using the Modified Monash Model (MMM) [43], based on the participant’s postcode. We used Poisson regression, with robust error sandwich estimators, to test for associations between respondent demographics (e.g., age, rurality and primary place of work as independent variables) and their degree of engagement in LARC and MA provision, with both their attitudes and knowledge (as dependent variables). Attitudes towards LARC and MA were defined as perceptions of safety, appropriateness, feasibility, and professional role; knowledge was defined as understanding of LARC and MA indications, contraindications, efficacy, and recommended timing. Results were reported as risk ratios (RR) and associated 95% confidence interval (CI). A *p*-value of < 0.05 was considered statistically significant. Text fields where ‘other’ responses were selected were grouped and analysed by key ideas.

### Ethics approval

Ethical approval was granted by the Monash University Human Research Ethics Committee (#28002) on 21 April 2021.

## Results

**Fig. 1:**
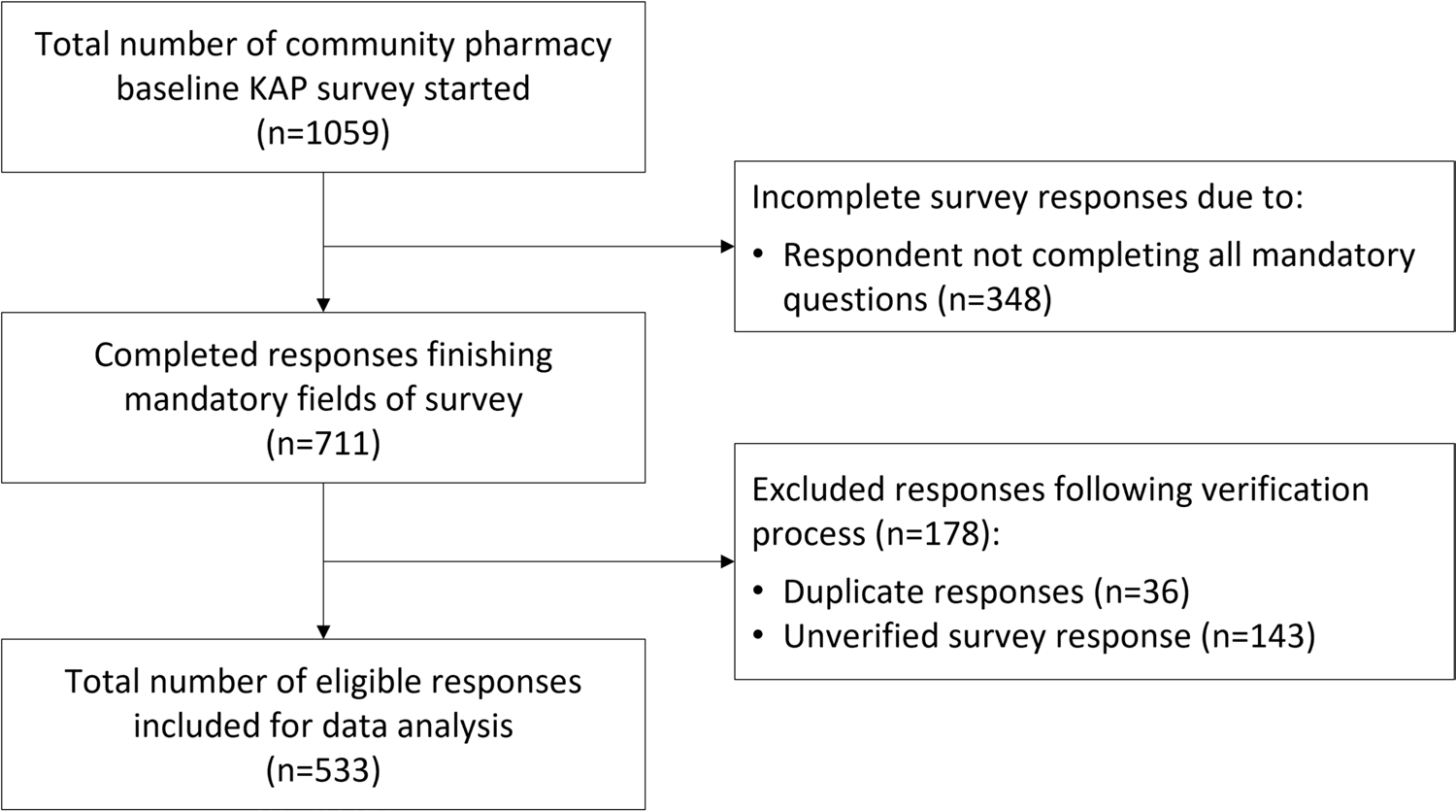
Response flow diagram

We received 1059 survey responses. After excluding incomplete mandatory responses (n = 348), duplicates (n = 36), and pharmacists who could not be verified against an active AHRPA registration number (n = 143), the final sample included 533 responses (Fig. 1).

### Pharmacist demographics

Most respondents identified as women (72.2%), were aged 31–40 (40%) and worked in metropolitan areas (70.9%) (Table 1). Over 45% had <10 years of community pharmacy experience. Compared to national health workforce data from the same year data was collected, the survey sample’s location of work (72.2% vs 74.2%) and years of experience (45% vs 33.6%) were similar [30].

**Table 1:** Demographic characteristics of community pharmacists (n = 533)

Characteristics	Baseline n (%)
	n = 533
Age group (years)	
≤ 30	148 (27.8)
31–40	213 (40.0)
41–50	83 (15.6)
51–60	56 (10.5)
> 60	33 (6.2)
Gender*	
Woman	385 (72.2)
Man	141 (26.5)
Other	7 (1.3)
Rurality^^^	
1 (Metropolitan)	378 (70.9)
2–5 (Regional/Rural)	134 (25.1)
6–7 (Remote/Very remote)	20 (3.8)
State^^^	
New South Wales	123 (23.1)
Victoria	208 (39.0)
Queensland	78 (14.6)
South Australia	39 (7.3)
Northern Territory	6 (1.1)
Western Australia	57 10.7)
Tasmania	8 (1.5)
Australian Capital Territory	13 (2.4)
Years worked in community pharmacy	
0–4	120 (22.5)
5–9	125 (23.5)
10–14	114 (21.4)
15–19	42 (7.9)
20–24	46 (8.6)
25–29	22 (4.1)
30+	64 (12.0)
Community pharmacy type	
Independent pharmacy	216 (40.5)
Banner group	276 (51.8)
Friendly society	30 (5.6)
Other e.g., General practice clinic, not stated	11 (2.1)
Qualifications held *(multiple choices allowed)*	
Bachelor of pharmacy	455 (85.4)
Masters of pharmacy	72 (13.5)
Overseas trained	15 (2.8)
Other	22 (4.1)
Community pharmcist role (*multiple choices allowed)*	
Intern	23 (4.3)
Pharmacist	228 (42.8)
Pharmacist in charge	186 (34.9)
Pharmacy manager	119 (22.3)
Pharmacy owner	72 (13.5)
Locum pharmacist	50 (9.4)
General practice pharmacist	10 (1.9)
Other	5 (0.9)
Consults in a language other than English	
No	397 (74.5)
Yes	136 (25.5)

*‘Other’ combines the remaining options, including ‘non-binary’, ‘my gender identity isn't listed’, and ‘prefer not to answer’

^^^With missing data, due to Modified Monash Model (MMM) yet to be classified postcodes or no response

### Pharmacists’ LARC knowledge, attitudes and practices

Respondents demonstrated high knowledge (Table 2). Most correctly identified LARC methods as more effective than oral contraceptive pills (88%), and acknowledged the influence of pharmacists' views and advice on patients’ contraceptive choices (83.9%). Only 66% knew that IUDs are suitable for nulliparous women (Table 2).

**Table 2:** Community pharmacists’ LARC knowledge, attitude and practice

Items	Baseline n (%)
Knowledge about LARC	n = 533
Long-acting reversible contraceptives are less effective than the contraceptive pill at preventing pregnancy *[Correct response—False]*	469 (88.0)
Intrauterine devices are suitable for use in nulliparous women *[Correct response—True]*	351 (65.9)
Pharmacists' views and advice can influence the type of contraception selected by patients *[Correct response—True]*	447 (83.9)
Fertility can return rapidly after long-acting reversible contraceptive removal *[Correct response—True]*	361 (67.7)
Attitudes about LARC	
Do you think the possible side effects of an intrauterine device outweigh the benefits?	
No	300 (56.3)
Yes	151 (28.3)
Unsure	82 (15.4)
Do you think the possible side effects of contraceptive implants outweigh the benefits?	
No	305 (57.2)
Yes	154 (28.9)
Unsure	74 (13.9)
Practice about LARC	
Service provision for intrauterine device	
Do you dispense intrauterine devices?	
Yes	495 (92.9)
No	38 (7.1)
How many intrauterine devices do you dispense in a typical month?	
< 1	72 (13.5)
1–5	394 (73.9)
> 5	67 (12.6)
Service provision for contraceptive implant	
Do you dispense implant?	
Yes	509 (95.5)
No	24 (4.5)
How many implants do you dispense in a typical month?	
< 1	55 (10.3)
1–5	403 (75.6)
> 5	75 (14.1)

*LARC* long-acting reversible contraception

Most respondents dispensed IUDs (92.9%) and implants (95.5%). Prescribers of LARC methods were commonly GPs (IUD: 69.6%; n = 371, implant: 80.0%; n = 421) and private gynaecologists (IUD: 34.1%; n = 182; implant: 23.2%; n = 124) (Table 2).

### Relationship between demographic characteristics and LARC knowledge, attitudes and practices

When comparing the relationship between respondents’ demographic characteristics and LARC dispensing (Table 3), pharmacists working in remote/very remote areas were 8% (RR 95% CI 1.08, 1.05–1.11) and 6% (RR 95% CI 1.06, 1.03–1.08) more likely to dispense IUDs and implants respectively.

**Table 3:** Relationship between community pharmacist characteristics and LARC practices

Characteristic	Practices			
	Dispense IUD		Dispense implant	
	Row n (%)	RRR (95% CI)	Row n (%)	RRR (95% CI)
Age				
< 30	140 (94.6)	REF	142 (95.9)	REF
30–39	201 (94.4)	0.99 (0.95–1.05)	208 (97.7)	1.02 (0.98–1.06)
40–49	75 (90.4)	0.96 (0.89–1.04)	80 (96.4)	1.01 (0.95–1.06)
50–59	51 (91.1)	0.96 (0.88–1.05)	52 (92.9)	0.96 (0.89–1.05)
60+	28 (84.9)	0.89 (0.77–1.04)	27 (81.8)	0.85 (0.72–1.01)
Gender				
Man	130 (92.2)	Ref	135 (95.7)	Ref
Woman	359 (93.3)	1.01 (0.96–1.07)	368 (95.6)	0.99 (0.96–1.04)
Rurality				
1 (Metropolitan)	350 (92.6)	Ref	358 (94.7)	Ref
2–5 (Regional/Rural)	124 (92.5)	0.99 (0.95–1.06)	130 (97.0)	1.02 (0.98–1.06)
6–7 (Remote/Very remote)	20 (100)	**1.08 (1.05–1.11)**	20 (100)	**1.06 (1.03–1.08)**
Year in community pharmacy				
0–4	117 (97.5)	Ref	119 (99.2)	Ref
5–9	113 (90.4)	**0.93 (0.87–0.99)**	118 (94.4)	**0.95 (0.91–0.99)**
10–14	108 (94.7)	0.97 (0.92–1.02)	110 (96.5)	0.97 (0.94–1.01)
15–19	38 (90.5)	0.93 (0.84–1.03)	39 (92.9)	0.94 (0.86–1.02)
20–24	40 (87.0)	0.89 (0.79–1.01)	44 (95.7)	0.96 (0.90–1.03)
25–29	21 (95.5)	0.98 (0.89–1.08)	21 (95.5)	0.96 (0.88–1.06)
30+	58 (90.6)	0.93 (0.85–1.01)	58 (90.6)	**0.91 (0.84–0.99)**
Community pharmacy type				
Independent pharmacy	198 (91.7)	Ref	207 (95.8)	Ref
Banner group	262 (94.9)	1.03 (0.99–1.09)	268 (97.1)	1.03 (0.98–1.04)
Friendly society	29 (96.9)	1.05 (0.98–1.14)	28 (93.3)	0.97 (0.88–1.07)
Other e.g., general practice clinic, not stated	6 (54.5)	0.60 (0.37–1.02)	6 (54.5)	**0.57 (0.33–0.98)**
Qualifications held				
Bachelor pharmacy				
No	70 (89.7)	Ref	72 (92.3)	Ref
Yes	425 (93.4)	1.04 (0.96–1.13)	437 (96.0)	1.04 (0.97–1.11)
Master pharmacy				
No	429 (93.1)	Ref	441 (95.6)	Ref
Yes	66 (91.7)	0.99 (0.91–1.06)	68 (94.4)	0.99 (0.93–1.05)
State				
New South Wales	113 (91.8)	Ref	117 (95.1)	Ref
Victoria	193 (92.8)	1.01 (0.95–1.08)	198 (95.2)	1.01 (0.95–1.05)
Queensland	72 (92.3)	1.01 (0.92–1.09)	75 (96.2)	1.01 (0.95–1.07)
South Australia	37 (94.9)	1.03 (0.94–1.13)	37 (94.9)	1.00 (0.91–1.08)
Western Australia	53 (92.9)	1.01 (0.93–1.11)	55 (96.5)	1.01 (0.95–1.08)
Tasmania/Northern Territory/Australian Capital Territory	26 (96.3)	1.05 (0.96–1.15)	36 (96.3)	1.01 (0.93–1.11)

Bolded results indicate significant findings at *p* < 0.05. *RRR*: relative risk reduction

Analysis of community pharmacists’ attitudes found that those working in regional/rural areas were more likely to disagree with the attitude statement that the possible side effects of IUDs (RR 95% CI 1.40, 1.21–1.62) and implants (RR 95% CI 1.39, 1.20–1.60) outweigh the benefits (Supplementary Table 1).

Age, years of practice, and type of pharmacy practice were significantly related to correct LARC knowledge (Supplementary Table 2). Respondents aged 40–49 (RR 95% CI 1.10, 1.01–1.20) and those with 15–19 years of community pharmacy experience (RR 95% CI 1.13, 1.03–1.26) were more likely to be aware of LARC effectiveness compared to oral contraceptive pills. Respondents aged 50–59 (RR 95% CI 1.37, 1.14–1.63) and those with >20 years of community pharmacy experience were more likely to correctly answer that fertility rapidly returns after LARC removal. No statistically significant relationship was found between respondents’ characteristics and knowledge about IUD suitability for nulliparous women.

### Pharmacists’ MA knowledge, attitudes and practices

Whilst 74.5% (n = 397) correctly answered that MA can be self-administered at home, fewer knew the gestational limit (49%) or the correct order of MA medicine administration (43%) (Table 4).

**Table 4:** Community pharmacists knowledge, attitude and practice about medication abortion

Knowledge about medication abortion	n = 533
In Australia, medication abortion is registered for use up to 9 weeks (63 days) of gestation *[Correct response—True]*	261 (49.0)
Efficacy of medication abortion is similar to that of surgical abortion *[Correct response—True]*	266 (49.9)
Misoprostol is administered before mifepristone *[Correct response—False]*	231 (43.3)
Medication abortion medicines can be self-administered at home *[Correct response—True]*	397 (74.5)
Attitudes about medication abortion	
I have the knowledge to counsel women about the process of medication abortion	
Agree	354 (66.4)
Disagree	142 (26.6)
Neither	37 (6.9)
I feel confident to dispense medication abortion medication	
Agree	355 (66.6)
Disagree	138 (25.9)
Neither	40 (7.5)
It is acceptable for pharmacists to dispense medication abortion medications	
Agree	486 (91.2)
Disagree	27 (5.1)
Neither	20 (3.8)
I think women need to know more about the availability of medication abortion	
Agree	505 (94.7)
Disagree	6 (1.1)
Neither	22 (4.1)
Practice about medication abortion	
Do you dispense medication abortion medicines (MS-2 Step)?	
Yes	160 (30)
No	373 (70)
Approximately how many times have you dispensed MS-2 Step in the last month?	*n* = *160*
0–2	135 (84.4)
3–5	17 (10.6)
> 5	8 (5.0)
Number of years of experience providing medication abortion	
< 1 years	63 (39.4)
> 1–2 years	35 (21.9)
> 2–3 years	13 (8.1)
> 3–4 years	20 (12.5)
> 4 years	29 (18.1)
When you are dispensing medication abortion medicines (MS-2 Step), do you discuss long-acting reversible contraceptive options with the patient	
Yes, IUDs	5 (3.1)
Yes, implants	2 (1.3)
Yes, IUDs and implants	58 (36.3)
Neither	95 (59.4)

Acceptability to dispense MA was high (91.2%), and most agreed that women need greater awareness of MA availability (94.7%). Around two-thirds of respondents identified having knowledge (66.4%) and confidence (66.6%) to dispense MA (Table 4). However, only 30% (n = 160) of respondents were registered MA dispensers (Table 4). More than 50% of respondents who dispensed had been doing so for ≤2 years. When dispensing MA, most respondents did not discuss LARC methods (59.4%, n = 95).

When asked about the benefits of providing MA in community pharmacy, most respondents identified reduced travel for women (77.3%), cost-effectiveness (63.3%), and continuity of care (62.5%) as key advantages (Supplementary Figure).

### Relationship between demographic characteristics and MA knowledge, attitudes and practices

Respondents in regional/rural areas (RR 95% CI 1.70, 1.30–2.21) and remote/very remote areas (RR 95% CI 2.24, 1.45–3.46) were more likely to be registered MA medicines dispensers compared to those in metropolitan areas (Table 5). Conversely, those consulting in a language additional to English were less likely to be registered MA dispensers (RR 95% CI 0.67, 0.48–0.95).

**Table 5:** Relationship between CP characteristics and MA dispensing

Characteristic	Registered dispenser of medication abortion medicines (MS-2 Step)	
	Row n (%)	RRR (95% CI)
Age		
< 30	43 (29.1)	REF
30–39	62 (29.1)	1.00 (0.72–1.40)
40–49	28 (33.7)	1.16 (0.79–1.72)
50–59	18 (32.1)	1.11 (0.70–1.75)
60+	9 (27.3)	0.94 (0.51–1.73)
Gender		
Man	36 (25.5)	Ref
Woman	121 (31.4)	1.23 (0.90–1.70)
Rurality		
1 (Metropolitan)	93 (24.6)	Ref
2–5 (Regional/Rural)	56 (41.8)	**1.70 (1.30–2.21)**
6–7 (Remote/Very remote)	11 (55.0)	**2.24 (1.45–3.46)**
Year in community pharmacy		
0–4	32 (26.7)	Ref
5–9	37 (29.6)	1.11 (0.74–1.66)
10–14	39 (34.2)	1.28 (0.87–1.90)
15–19	14 (33.3)	1.25 (0.74–2.10)
20–24	12 (26.1)	0.98 (0.55–1.73)
25–29	6 (27.3)	1.02 (0.49–2.15)
30+	20 (31.3)	1.18 (0.73–1.87)
Community pharmacy type		
Independent pharmacy	52 (24.1)	Ref
Banner group	94 (34.1)	**1.41 (1.06–1.89)**
Friendly society	11 (36.7)	1.52 (0.90–2.58)
Other e.g., general practice clinic, not stated	3 (27.8)	1.13 (0.42–3.06)
Qualifications held		
BPharm		
No	24 (33.8)	Ref
Yes	136 (29.9)	0.97 (0.68–1.40)
MPharm		
No	136 (29.5)	Ref
Yes	24 (33.3)	1.13 (0.79–1.61)
State		
New South Wales	35 (28.5)	Ref
Victoria	60 (28.9)	1.01 (0.71–1.44)
Queensland	28 (35.9)	1.26 (0.84–1.90)
South Australia	12 (30.8)	1.08 (0.63–1.87)
Western Australia	14 (24.6)	0.86 (0.51–1.47)
Tasmania/Northern Territory/Australian Capital Territory	11 (40.7)	1.43 (0.84–2.44)
Consults in a language other than English		
No	130 (32.8)	Ref
Yes	30 (22.1)	**0.67 (0.48–0.95)**

Bolded results indicate significant findings at *p* < 0.05

Analysis of community pharmacists’ attitude towards MA found that those aged ≥60 years were more likely to indicate having the knowledge to counsel women (RR 1.29) and the confidence (RR 1.38) to dispense MA medicines (Supplementary Table 3). Respondents in remote/very remote areas showed higher agreement with the attitudinal statements regarding knowledge (*I have the knowledge to counsel women about the process of MA*) (RR 1.32) and confidence *(I feel confident to dispense MA medications) *(RR 1.39) to provide MA (Supplementary Table 3). Respondents with 15–24 years of community pharmacy experience and/or who were registered MA medication dispensers were also more likely to agree with both attitude statements (Supplementary Table 3).

Registered MA medication dispensers were more likely to answer all knowledge statements correctly. Respondents aged 50–59 (RR 95% CI 1.48, 1.14–1.92) and those with >30 years of experience in a community pharmacy (RR 95% CI 1.44, 1.09–1.91) were more likely to be aware of MA efficacy being similar to surgical abortion (Supplementary Table 4).

## Discussion

This study found that pharmacists possess good LARC knowledge, with pharmacists aged 40–49 and those with more extensive experience demonstrating more accurate knowledge of LARC effectiveness. Registered MA pharmacists were more likely to live in remote and very remote areas. Over half of registered pharmacists reported not routinely discussing LARC options during MA consultations. Study pharmacists, who speak a language other than English at home (5.6%) were less likely to be registered MA dispensers, raising concerns for linguistically diverse communities, given that 22% of Australians speak a language other than English at home [44].

Providing accurate, high-quality contraceptive method information, including efficacy, side effects, safety and cost, is essential, as improved access to information and methods reduces unintended pregnancy and abortion rates [45]. LARC uptake post-abortion reduces the likelihood of repeat MA [46]. Our findings build on Buckingham et al. [34], who reported that one-third of Australian community pharmacists receive weekly contraceptive advice requests from patients. However, pharmacists felt more confident with oral contraceptive pills than LARC methods, particularly IUDs [34]. Barriers to contraceptive counselling included insufficient training, resources and/or inadequate remuneration [34]. Similarly, Dev et al. [47], interviewed women regarding pharmacist-led provision of oral contraceptive pills, revealing that women perceived community pharmacies as convenient access points for oral contraceptive pills, with an expectation for pharmacists to be actively involved in delivering comprehensive contraceptive care.

Additional training and support are required for new MA dispensers, particularly since the registration requirements were removed in Australia in 2023 [48], along with the inclusion of training in delivering effectiveness-based contraceptive counselling, including LARC methods. However, sexual and reproductive health content in pharmacy university curricula remains limited [49]. Expanding MA and comprehensive effectiveness-based contraceptive counselling education in undergraduate and continuing education programs is critical to ensure evidence-based, patient-centred care. Similar regulatory changes, such as over-the-counter emergency contraceptive pills and pharmacist prescribing, have similarly occurred in other high-income countries [20, 50–52] highlighting the need for robust training and support. Targeted training modules in contraceptive prescribing would address pharmacist-identified barriers [53] such as knowledge gaps, comfort, and attitudes regarding pharmacists’ expanding sexual and reproductive health roles [50, 54].

Despite evidence supporting IUD safety and appropriateness for nulliparous women, misconceptions persist among pharmacists [55]. This survey found no statistically significant relationship between pharmacists’ demographic characteristics and correct responses regarding nulliparity and IUDs. Older and more experienced community pharmacists were associated with greater LARC knowledge, including its higher efficacy compared to oral contraceptive pills and rapid return of fertility post-removal. These results echo findings from US studies, where 20–30% of obstetrician-gynaecologists, physicians, nurses, and midwives had misconceptions about IUDs suitability for nulliparous women, due to perceived insertion challenges due to the cervices not stretching previously and safety concerns. However, these misconceptions decreased with age, training and familiarity [55–58]. This suggests a training gap among newer pharmacists, reinforcing the need for improved undergraduate and ongoing education on LARC and MA, to enhance pharmacists’ knowledge and competency in practice [59]. In this study, non-metropolitan pharmacists reported greater confidence in MA counselling and dispensing, likely due to greater exposure. Continuous exposure and targeted training improve pharmacists’ knowledge, attitudes, and comfort as these services integrate into everyday practice [49, 50]. Women rely on healthcare professionals' endorsement when choosing IUDs [60, 61]. Given the influence of non-expert opinions, often based on personal or peer experiences (the latter of which may be negative or inaccurate) [61, 62], pharmacists' need to be equipped and competent to provide information on IUDs, particularly for nulliparous women [63].

‘Abortion deserts’ describe areas lacking abortion services within the public and private healthcare system [64] or an area where a person lives 160 km from the nearest known abortion service [65]. In Australia, approximately 25% of women aged 15–54 live without a local MA prescriber or dispenser [64]. A recent study examining abortion referral pathways across Australian primary health networks found that only half of the reviewed networks had listed publicly funded surgical abortion referral services [66]. Limited regional services have driven reliance on telehealth, increasing demand for MA dispensers in regional/rural, and remote/very remote areas. Our findings reflect this, with pharmacists in remote/very remote areas more likely to dispense MA and LARC. This demand for registered MA dispensers outside of metropolitan areas may be driven by the lack of primary care and hospital-based surgical abortion providers [67], and private surgical providers mainly located in metropolitan areas [68]. Community pharmacists in rural areas are supporting community needs, working alongside family physicians who prescribe MA medications in rural practice or via telehealth. Telehealth partially addresses these gaps, but local MA dispensing remains essential for equitable access.

### Strengths/limitations

This is the only Australian study to examine community pharmacists’ knowledge, attitudes and practices regarding LARC and MA. A higher proportion of respondents were registered MA dispensers compared with national statistics at the time of this study, likely reflecting respondents’ greater interest in this area than other community pharmacists, and their willingness to take part. However, respondents' demographic characteristics were largely representative of community pharmacists nationally, with a higher proportion working in metropolitan areas, being female, aged in their 20–30 s, and with ≤10 years of community pharmacy experience. This survey had a higher proportion of female respondents (72.2%) than nationally in 2021(56%; n = 9545) [30], though it aligned more closely with the general pharmacy workforce, where 62.4% of pharmacists are women [30]. Survey fraud is increasingly an issue for researchers [69]. In this study, 178 respondents could not be verified or were duplicates, likely influenced by the financial incentive offered. A key strength was our use of robust fraud detection and mitigation strategies, reported elsewhere. Electronic and human verification methods were used to detect and remove fraudulent data, supporting data validity.

## Conclusion

This study provides important insights into Australian community pharmacists’ knowledge, attitudes, and practices regarding LARC and MA. While pharmacists demonstrated baseline knowledge and willingness to engage in contraceptive care, knowledge gaps among younger pharmacists suggest gaps in undergraduate curricula. Registered pharmacists exhibited more favourable attitudes and greater confidence in MA counselling and dispensing, particularly in rural and remote areas with limited surgical abortion access. However, many pharmacists did not routinely discuss LARC options during MA consultations, missing opportunities for integrated contraceptive care. The removal of MA registration requirements further underscores the urgency of embedding comprehensive, evidence-based training on MA and LARC into both undergraduate and continuing professional education. Addressing structural barriers such as limited training opportunities, inadequate remuneration, and insufficient access to clinical resources is essential to enable pharmacists to deliver high-quality, patient-centred sexual and reproductive health services. Strengthening these supports is critical to ensuring pharmacists are well-equipped to meet the growing demand for accessible, equitable reproductive healthcare across diverse practice settings.

## Supplementary Information

Below is the link to the electronic supplementary material.

Supplementary file1 (PDF 576 KB)

## Data Availability

The dataset generated and analysed during the current study is not available.
