# Supplementary material for: Long-acting reversible contraception and medication abortion: a national descriptive survey of Australian community pharmacist knowledge, attitudes and practices
Source: Int J Clin Pharm. 2026 Feb 24;48(3):935–48. doi: 10.1007/s11096-026-02088-1 (PMC13176050; doi:10.1007/s11096-026-02088-1)

Supplementary File 1 - AusCAPPS Baseline Pharmacy Survey

Data Dictionary Codebook

AusCAPPS Baseline_Pharm

#	Variable / Field Name	Field Label<br>*Field Note*	Field Attributes (Field Type, Validation, Choices, Calculations, etc.)
**Instrument: Into Question (into_question)** Enabled as survey			
1	[record_id]	Record ID	text
2	[intro]	We are inviting you to take part in a short knowledge, attitudes, and current practice survey about long-acting reversible contraception (e.g. intrauterine devices and contraceptive implants) and medical abortion in primary care (Note: Medical abortion is defined as using mifepristone followed by misoprostol to end an early pregnancy up to 63 days) It should take no more than 15 minutes to complete. You will receive a $40 gift voucher upon completion and verification via the email that you provide.Please read the attached Explanatory Statement in full before deciding whether or not to participate in this research.	descriptive<br>(Attachment: Monash Ethics Explanatory Statement_KAP Survey.pdf, Display format: Link), Required
3	[honeypot]	Please click yes to continue:	yesno, Required<br>1 Yes<br>0 No<br><br>Field Annotation: @HIDDEN-SURVEY<br>Stop actions on 1, 0
4	[practitioner]	Please select your practitioner group	radio, Required<br>1 General Practitioner<br>2 Nurse/Nurse practitioner<br>3 Community pharmacist
5	[into_question_complete]	Section Header: *Form Status*<br>Complete?	dropdown<br>0 Incomplete<br>1 Unverified<br>2 Complete

**Instrument: KAP Survey Pharmacist (kap_survey_pharmacist)** Enabled as survey			
166	[primarycare_pharm]	Do you work in primary care?	yesno

			1 Yes<br>0 No<br><br>Stop actions on 0
167	[timestamp_pharm]	Section Header: *SECTION 1: DEMOGRAPHICS*<br>timestamp	text (datetime_seconds_dmy)<br>Field Annotation: @HIDDEN @NOW
168	[yearsinprimarycare_v3]	How many years have you worked in primary care?	text (number), Required
169	[postcodes_v3]	Practice Postcode: Primary pharmacy post code {primary_postcode_v3} Secondary pharmacy post code (if applicable) {secondary_postcode_v3}	descriptive
170	[primary_postcode_v3]	Primary practice postcode	text (postalcode_australia), Required
171	[secondary_postcode_v3]	Secondary practice postcode<br>(if applicable)	text (postalcode_australia)
172	[gender_v3]	Gender	checkbox, Required<br>1 gender_v3___1 Male or man<br>2 gender_v3___2 Female or woman<br>3 gender_v3___3 Non-binary<br>4 gender_v3___4 My gender identity isn't listed. I identify as: {gender_other_v3}<br>5 gender_v3___5 Prefer not to answer
173	[gender_other_v3]	other	text (alpha_only)
174	[age_v3]	Age (years)	radio, Required<br>1 18-24<br>2 25-29<br>3 30-34<br>4 35-39<br>5 40-44<br>6 45-49<br>7 50-54<br>8 55-59<br>9 60-64<br>10 65+
175	[qualification_v3]	Please select the qualification(s) relevant to you:	checkbox, Required<br>1 qualification_v3___1 BPharm<br>2 qualification_v3___2 MPharm<br>3 qualification_v3___3 Overseas Training<br>4 qualification_v3___4 Other {qualification_othe
176	[qualification_other_v3]	Other	text
177	[role_v3]	What is your role in the pharmacy? Please tick all that apply	checkbox, Required<br>1 role_v3___1 Intern pharmacist<br>2 role_v3___2 Pharmacist

			3 role_v3___3 Pharmacist in charge<br>4 role_v3___4 Pharmacy manager<br>5 role_v3___5 Pharmacy owner<br>6 role_v3___6 Locum pharmacist<br>7 role_v3___7 General practice pharmacist<br>8 role_v3___8 Other {roleother_v3}
178	[roleother_v3]	other	text (alpha_only)
179	[primarypractice_type_v3]	Which of the following best describes the type of community pharmacy in which you work?	radio, Required<br>1 Independent pharmacy<br>2 Banner group<br>3 Friendly society<br>4 Other {primarypractice_type_other_v3}
180	[primarypractice_type_other_v3]		text
181	[consultlanguage_v3]	Do you conduct your consultations in a language other than English?	radio, Required<br>1 Yes {other_language_v3}<br>0 No
182	[other_language_v3]<br>Show the field ONLY if:<br>[consultlanguage_v3]=1	Other	text
183	[q1_v3]	Section Header: *SECTION 2: PRACTICES*<br>Please answer the below questions about Intrauterine devices and contraceptive implants in your pharmacy: Intrauterine devices Contraceptive implants Do you dispense? {q1a_iud_v3} {q1a_implants_v3} How many do you dispense in a typical month? (Please enter '0' if you do not dispense) {q1b_iud_v3} {q1b_implants_v3} Where do your patients typically have their insertions? Please tick all that apply. {q1d_iud_v3} {q1d_implants_v3}	descriptive
184	[q1a_iud_v3]	Do you dispense?	yesno, Required<br>1 Yes<br>0 No
185	[q1a_implants_v3]	Do you dispense?	yesno, Required<br>1 Yes<br>0 No
186	[q1b_iud_v3]	How many have you dispense in the last month?	text (number), Required
187	[q1b_implants_v3]	How many have you dispense in the last month?	text (number), Required
188	[q1d_iud_v3]	Where do women you consult have their insertions?	checkbox, Required<br>1 q1d_iud_v3___1 Private gynaecologist<br>2 q1d_iud_v3___2 Public hospital

			3 | q1d_iud_v3___3 | Family planning clinic<br>4 | q1d_iud_v3___4 | Sexual health clinic<br>5 | q1d_iud_v3___5 | A GP<br>6 | q1d_iud_v3___6 | Nurse practitioner<br>8 | q1d_iud_v3___8 | Unknown/not sure
189	[q1d_implants_v3]	Where do women you consult have their insertions?	checkbox, Required<br>1 | q1d_implants_v3___1 | Private gynaecologist<br>2 | q1d_implants_v3___2 | Public hospital<br>3 | q1d_implants_v3___3 | Family planning clinic<br>4 | q1d_implants_v3___4 | Sexual health clinic<br>5 | q1d_implants_v3___5 | A GP<br>6 | q1d_implants_v3___6 | Nurse practitioner<br>7 | q1d_implants_v3___7 | Unknown/not sure
190	[q2_v3]	How often would you initiate discussions about long-acting reversible contraceptives (e.g. intrauterine devices and contraceptive implants) in your contraceptive consultations?	radio, Required<br>1 | Never<br>2 | Rarely<br>3 | Sometimes<br>4 | Very often<br>5 | Always
191	[verification_text2]	What impact has COVID-19 had on your long-acting reversible contraception service provision?	text, Required
192	[q3a_v3]	Are you an accredited dispenser of medical abortion medicines (MS-2 Step)?	yesno, Required<br>1 | Yes<br>0 | No
193	[q3b_v3]<br>Show the field ONLY if:<br>[q3a_v3]=1	How many years of experience do you have providing medical abortion? (years)	text (number), Required
194	[q3c_v3]<br>Show the field ONLY if:<br>[q3a_v3]=1	Approximately how many medical abortions do you provide in a typical month?	text (number), Required
195	[q3d_v3]<br>Show the field ONLY if:<br>[q3a_v3]=1	Who is aware of your work as a medical abortion provider? Please tick all that apply.	checkbox, Required<br>1 | q3d_v3___1 | A local pharmacist<br>2 | q3d_v3___2 | A local GP<br>3 | q3d_v3___3 | Other local health professionals<br>4 | q3d_v3___4 | My practice manager/receptionist

			5 | q3d_v3___5 | Other GPs in my practice<br>6 | q3d_v3___6 | Practice nurses in my practice<br>7 | q3d_v3___7 | The local radiology practice(s)<br>8 | q3d_v3___8 | The local pathology provider(s)<br>9 | q3d_v3___9 | A local gynaecologist<br>10 | q3d_v3___10 | My local emergency department<br>11 | q3d_v3___11 | Other {q3d_other_v3}
196	[q3d_other_v3]	Other	text
197	[q4_v3]	In the pharmacy that you work, are there other pharmacists accredited to dispense medical abortion medicines (MS-2 Step)?	radio, Required<br>1 | Yes (please specify how many) {q4count}<br>0 | No
198	[q4count]	How many	text (number)
199	[q4a_v3]<br>Show the field ONLY if:<br>[q4_v3]=0	Do you have colleagues who you can refer to dispense medical abortion medicines (MS-2 Step)?	yesno, Required<br>1 | Yes<br>0 | No
200	[q5_v3]	Do you have anyone to whom you can refer for medical abortion medicines if required?	checkbox, Required<br>1 | q5_v3___1 | Private gynaecologist<br>2 | q5_v3___2 | Public hospital<br>3 | q5_v3___3 | Family planning clinic<br>4 | q5_v3___4 | A GP<br>5 | q5_v3___5 | Private abortion service<br>6 | q5_v3___6 | Unknown/not sure
201	[q6_v3]	When you are dispensing medical abortion medicines (MS-2 Step), do you discuss long-acting reversible contraceptive options with the patient?	checkbox, Required<br>1 | q6_v3___1 | Yes, IUDs<br>2 | q6_v3___2 | Yes, implants<br>3 | q6_v3___3 | Yes, IUDs and implants<br>4 | q6_v3___4 | Neither
202	[q7a_v3]	Section Header: *SECTION 3: KNOWLEDGE Please indicate your agreement with the following statements:*<br>Long-acting reversible contraceptives are less effective than the contraceptive pill at preventing pregnancy	radio (Matrix), Required<br>1 | True<br>2 | False<br>3 | Unsure
203	[q7b_v3]	Intrauterine devices' are suitable for use in nulliparous women	radio (Matrix), Required<br>1 | True<br>2 | False<br>3 | Unsure
204	[q7c_v3]	Pharmacists' views and advice can influence the type of contraception selected by patients	radio (Matrix), Required<br>1 | True

			2 False<br>3 Unsure
205	[q7d_v3]	Fertility can return rapidly after long-acting reversible contraceptive removal	radio (Matrix), Required<br>1 True<br>2 False<br>3 Unsure
206	[q7e_v3]	In Australia, medical abortion is registered for use up to 9 weeks (63 days) gestation in all states	radio (Matrix), Required<br>1 True<br>2 False<br>3 Unsure
207	[q7f_v3]	Efficacy of medical abortion is similar to that of surgical abortion	radio (Matrix), Required<br>1 True<br>2 False<br>3 Unsure
208	[q7g_v3]	Misoprostol is administered before mifepristone	radio (Matrix), Required<br>1 True<br>2 False<br>3 Unsure
209	[qh_v3]	Medical abortion medicines can be self-administered at home	radio (Matrix), Required<br>1 True<br>2 False<br>3 Unsure
210	[sideeffectabortion_v3]	What are the most common side effects from medical abortion? Please tick all that apply.	checkbox, Required<br>1 sideeffectabortion_v3___1 Bleeding<br>2 sideeffectabortion_v3___2 Cramping<br>3 sideeffectabortion_v3___3 Nausea/vom[illegible]<br>4 sideeffectabortion_v3___4 Thrombocyt[illegible]<br>5 sideeffectabortion_v3___5 Fever/chills<br>6 sideeffectabortion_v3___6 All of the ab[illegible]<br>7 sideeffectabortion_v3___7 Don't know<br><br>Field Annotation: @NONEOFTHEABOVE=6
211	[q9a_v3]	Section Header: *SECTION 4: ATTITUDES Please indicate your agreement with the following statements:*<br><br>I have the knowledge to counsel women about the process of medical abortion	radio (Matrix), Required<br>1 Agree<br>2 Disagree<br>3 Neither
212	[q9b_v3]	I feel confident to dispense medical abortion medications	radio (Matrix), Required<br>1 Agree<br>2 Disagree<br>3 Neither
213	[q9c_v3]	It is acceptable for pharmacists to dispense medical abortion medications	radio (Matrix), Required<br>1 Agree

			2 Disagree<br>3 Neither
214	[q9d_v3]	I think women need to know more about the availability of medical abortion	radio (Matrix), Required<br>1 Agree<br>2 Disagree<br>3 Neither
215	[q10_v3]	Do you think the possible side effects of intrauterine devices outweigh the benefits?	radio, Required<br>1 Yes<br>2 Unsure<br>0 No
216	[q11_v3]	Do you think the possible side effects of contraceptive implants outweigh the benefits?	radio, Required<br>1 Yes<br>2 Unsure<br>0 No
217	[q12_v3]	What factors influence you to recommend long-acting reversible contraceptives to an eligible patient? Please tick all that apply.	checkbox, Required<br>1 q12_v3___1 Age<br>2 q12_v3___2 Cost<br>3 q12_v3___3 Patient's BMI<br>4 q12_v3___4 Past history of abnormal cytology<br>5 q12_v3___5 Past history of sexually transmitted infections<br>6 q12_v3___6 Marital status<br>7 q12_v3___7 History of full-term pregnancies<br>8 q12_v3___8 History of abortion<br>9 q12_v3___9 I don't not recommend long-acting reversible contraceptives<br>10 q12_v3___10 Other {q12_other_v3}
218	[q12_other_v3]	other	text
219	[q13_v3]	What would dissuade you from dispensing/counselling on medical abortion to an eligible patient? Please tick all that apply.	checkbox, Required<br>1 q13_v3___1 I don't feel I have the knowledge to recommend MS-2 Step<br>2 q13_v3___2 I don't feel I have the skills to recommend MS-2 Step<br>3 q13_v3___3 Cost to patient<br>4 q13_v3___4 Concerns about safety<br>5 q13_v3___5 Concerns about efficacy

			6 | q13_v3___6 | I am afraid of the stigma associated with dispensing medical abortion<br>7 | q13_v3___7 | Lack of referral options to specialists/hospitals if a complication occurs<br>8 | q13_v3___8 | Previous negative experience supporting a patient undergoing medical abortion<br>9 | q13_v3___9 | I am a conscientious objector to termination of pregnancy<br>10 | q13_v3___10 | Lack of confidential space<br>11 | q13_v3___11 | Lack of time<br>12 | q13_v3___12 | None<br>13 | q13_v3___13 | Other {q13_other_v3}<br><br>Field Annotation: @NONEOFTHEABOVE=12
220	[q13_other_v3]	other	text
221	[q14_v3]	What are the benefits of dispensing medical abortion medicines in a pharmacy? Please tick all that apply.	checkbox, Required<br>1 | q14_v3___1 | It is a cost-effective option for women<br>2 | q14_v3___2 | Opportunity to provide more comprehensive care<br>3 | q14_v3___3 | Offers continuity of care for women<br>4 | q14_v3___4 | Reduces women's need to travel to access an abortion services<br>5 | q14_v3___5 | Increased confidentiality for the woman<br>6 | q14_v3___6 | Contraceptive care can be delivered at the same time<br>7 | q14_v3___7 | I can't see any benefit<br>8 | q14_v3___8 | Other {q14_other_v3}
222	[q14_other_v3]	other	text
223	[av3]	Section Header: *SECTION 5: EXPERIENCES Please indicate which option most closely reflects your experience*<br><br>Media take a balanced view on medical abortion	radio (Matrix), Required<br>1 | Strongly disagree<br>2 | Disagree<br>3 | Neither

			4 Agree<br>5 Strongly agree<br>6 NA
224	[bv3]	I feel discriminated against by other healthcare professionals because of my decision to provide medical abortion services	radio (Matrix), Required<br>1 Strongly disagree<br>2 Disagree<br>3 Neither<br>4 Agree<br>5 Strongly agree<br>6 NA
225	[cv3]	I am proud that I work in abortion care	radio (Matrix), Required<br>1 Strongly disagree<br>2 Disagree<br>3 Neither<br>4 Agree<br>5 Strongly agree<br>6 NA
226	[dv3]	I feel connected to others who do this work	radio (Matrix), Required<br>1 Strongly disagree<br>2 Disagree<br>3 Neither<br>4 Agree<br>5 Strongly agree<br>6 NA
227	[ev3]	I feel that society appreciates the work I do in abortion care	radio (Matrix), Required<br>1 Strongly disagree<br>2 Disagree<br>3 Neither<br>4 Agree<br>5 Strongly agree<br>6 NA
228	[fv3]	I am afraid that if I tell people I work in abortion care I could put myself or my loved ones at risk of harassment/violence	radio (Matrix), Required<br>1 Strongly disagree<br>2 Disagree<br>3 Neither<br>4 Agree<br>5 Strongly agree<br>6 NA
229	[gv3]	I have never experienced harassment and/or violence as a result of working in abortion care	radio (Matrix), Required<br>1 Strongly disagree<br>2 Disagree<br>3 Neither

			4 Agree<br>5 Strongly agree<br>6 NA
230	[hv3]	I did not realise my concerns with dispensing before becoming accredited were not realised once I became accredited	radio (Matrix), Required<br>1 Strongly disagree<br>2 Disagree<br>3 Neither<br>4 Agree<br>5 Strongly agree<br>6 NA
231	[iv3]	I feel confident to dispense medical abortion medicine	radio (Matrix), Required<br>1 Strongly disagree<br>2 Disagree<br>3 Neither<br>4 Agree<br>5 Strongly agree<br>6 NA
232	[kap_survey_pharmacist_complete]	Section Header: *Form Status*<br>Complete?	dropdown<br>0 Incomplete<br>1 Unverified<br>2 Complete

Supplementary Table 1. Relationship between CP characteristics and attitude towards benefits of LARC

Characteristic	**Attitude -** Disagree with the following statements: *"The possible side effects of … outweigh the benefits"*			
	**IUD**		**Implant**	
	**Row n (%)**	**RRR (95% CI)**	**Row n (%)**	**RRR (95% CI)**
**Age**				
<30	85 (57.4)	REF	85 (57.4)	REF
30 – 39	118 (55.4)	0.96 (0.80 – 1.16)	120 (56.3)	0.98 (0.82 – 1.18)
40 – 49	48 (57.8)	1.01 (0.80 – 1.27)	49 (59.0)	1.03 (0.82 – 1.23)
50 – 59	31 (55.7)	0.96 (0.73 – 1.27)	30 (53.6)	0.93 (0.70 – 1.34)
60+	18 (54.6)	0.95 (0.67 – 1.34)	21 (63.6)	1.11 (0.83 – 1.49)
**Gender**				
Man	78 (55.3)	Ref	81 (57.5)	Ref
Woman	219 (56.9)	1.02 (0.87 – 1.22)	220 (57.1)	0.99 (0.84 – 1.17)
**Rurality**				
1 (Metropolitan)	193 (51.1)	Ref	197 (52.1)	Ref
2-5 (Regional/Rural)	96 (71.6)	**1.40 (1.21 – 1.62)**	97 (72.4)	**1.39 (1.20 – 1.60)**
6-7 (Remote/Very Remote)	11 (55.0)	1.08 (0.72 – 1.62)	11 (55.0)	1.05 (0.71 – 1.59)
**Year in community pharmacy**				
0-4	69 (57.5)	Ref	50 (58.3)	Ref
5-9	69 (52.0)	0.91 (0.72 – 1.14)	65 (52.0)	0.89 (0.71 – 1.12)
10-14	66 (57.9)	1.01 (0.81 – 1.25)	69 (60.5)	1.04 (0.84 – 1.28)
15-19	22 (52.4)	0.91 (0.66 – 1.26)	20 (47.6)	0.82 (0.57 – 1.16)
20-24	27 (58.7)	1.02 (0.77 – 1.36)	27 (58.7)	1.01 (0.76 – 1.34)
25-29	15 (68.2)	1.19 (0.86 – 1.64)	15 (68.2)	1.17 (0.85 – 1.62)
30+	36 (56.3)	0.98 (0.75 – 1.28)	39 (60.9)	1.04 (0.82 – 1.34)
**Community Pharmacy type**				
Independent Pharmacy	118 (54.6)	Ref	121 (56.0)	Ref
Banner Group	160 (58.0)	1.06 (0.91 – 1.24)	162 (58.7)	1.05 (0.88 – 1.22)
Friendly Society	16 (53.3)	0.98 (0.68 – 1.40)	16 (53.3)	0.95 (0.68 – 1.36)
Other e.g., GP clinic, not stated	6 (54.5)	0.99 (0.57 – 1.74)	6 (54.5)	0.97 (0.56 – 1.69)
**Qualifications held**				
BPharm				
No	37 (47.4)	Ref	38 (48.7)	Ref
Yes	263 (57.8)	1.22 (0.95 – 1.56)	267 (58.7)	1.20 (0.95 – 1.53)
MPharm				
No	262 (56.8)	Ref	266 (57.7)	Ref
Yes	38 (52.8)	0.93 (0.74 – 1.17)	39 (54.2)	0.94 (0.75 – 1.18)

Note: Bolded results indicate significant findings at p < 0.05.

Supplementary Table 2. Relationship between CP characteristics and LARC Knowledge

Characteristic	Knowledge Statements					
	Long-acting reversible contraceptives are less effective than the contraceptive pill at preventing pregnancy (False)		Intrauterine devices are suitable for use in nulliparous women (True)		Fertility can return rapidly after long-acting reversible contraceptive removal (True)	
	**Row n (%)**	**RRR (95% CI)**	**Row n (%)**	**RRR (95% CI)**	**Row n (%)**	**RRR (95% CI)**
**Age**						
<30	125 (84.5)	REF	88 (59.5)	REF	89 (60.1)	REF
30 – 39	190 (89.2)	1.06 (0.97 – 1.15)	148 (69.5)	1.17 (0.99 – 1.37)	144 (67.6)	1.12 (0.96 – 1.32)
40 – 49	77 (92.8)	**1.10 (1.01 – 1.20)**	54 (65.1)	1.09 (0.89 – 1.34)	59 (71.1)	1.18 (0.98 – 1.43)
50 – 59	49 (87.5)	1.04 (0.92 – 1.17)	38 (67.9)	1.14 (0.91 – 1.43)	46 (82.1)	**1.37 (1.14 – 1.63)**
60+	28 (84.5)	1.00 (0.86 – 1.18)	23 (69.7)	1.17 (0.90 – 1.52)	23 (69.7)	1.16 (0.89 – 1.50)
**Gender**						
Man	119 (84.4)	Ref	88 (62.4)	Ref	94 (66.7)	Ref
Woman	344 (89.4)	1.06 (0.98 – 1.15)	260 (67.5)	1.08 (0.93 – 1.25)	264 (68.6)	1.03 (0.90 – 1.18)
**Rurality**						
1 (Metropolitan)	330 (87.3)	Ref	246 (65.1)	Ref	252 (66.7)	Ref
2-5 (Regional/Rural)	121 (90.3)	1.03 (0.97 – 1.11)	988(65.7)	1.01 (0.87 – 1.16)	95 (70.9)	1.06 (0.93 – 1.21)
6-7 (Remote/Very Remote)	17 (85.0)	1.08 (0.72 – 1.62)	16 (80.0)	1.22 (0.98 – 1.54)	13 (65.0)	0.98 (0.70 – 1.36)
**Year in Community Pharmacy**						
0-4	104 (86.7)	Ref	81 (67.5)	Ref	75 (62.5)	Ref
5-9	104 (83.2)	0.96 (0.86 – 1.07)	76 (60.8)	0.90 (0.75 – 1.09)	79 (63.2)	1.01 (0.83 – 1.23)
10-14	103 (90.4)	1.04 (0.95 – 1.14)	77 (67.5)	1.00 (0.84 – 1.19)	73 (64.0)	1.02 (0.84 – 1.25)
15-19	41 (97.6)	**1.13 (1.03 – 1.26)**	32 (76.2)	1.13 (0.91 – 1.39)	32 (76.2)	1.22 (0.98 – 1.52)
20-24	41 (89.1)	1.02 (0.91 – 1.16)	27 (58.7)	0.87 (0.66 – 1.14)	36 (78.3)	**1.25 (1.02 – 1.54)**
25-29	21 (95.5)	1.10 (0.98 – 1.24)	14 (63.6)	0.94 (0.67 – 1.32)	17 (77.3)	1.24 (0.95 – 1.61)
30+	55 (85.9)	0.99 (0.88 – 1.12)	44 (68.9)	1.02 (0.83 – 1.25)	49 (75.6)	**1.23 (1.01 – 1.49)**
**Community Pharmacy type**						
Independent Pharmacy	191 (88.4)	Ref	142 (65.7)	Ref	158 (73.2)	Ref
Banner Group	1242 (87.8)	0.99 (0.93 – 1.06)	183 (66.3)	1.01 (0.89 – 1.15)	176 (63.8)	**0.87 (0.77 – 0.98)**
Friendly Society	25 (83.3)	0.94 (0.80 – 1.11)	18 (60.0)	0.91 (0.67 – 1.24)	17 (56.7)	0.77 (0.56 – 1.07)
Other e.g., GP clinic, not stated	11 (100.0)	**1.13 (1.08 – 1.19)**	8 (72.7)	1.11 (0.76 – 1.61)	10 (90.9)	**1.24 (1.01 – 1.52)**
**Qualifications held**						
BPharm						
No	79 (89.7)	Ref	50 (64.1)	Ref	56 (71.8)	Ref
Yes	399 (87.7)	0.98 (0.90 – 1.06)	301 (66.1)	1.03 (0.86 – 1.23)	305 (67.0)	0.93 (0.80 – 1.09)
MPharm						
No	404 (87.6)	Ref	303 (65.7)	Ref	311(67.5)	Ref
Yes	65 (90.3)	1.03 (0.95 – 1.12)	48 (66.8)	1.01 (0.85 – 1.21)	50 (69.4)	1.03 (0.87 – 1.22)

Note: Bolded results indicate significant findings at $p < 0.05$.

Supplementary Table 3. Relationship between CP characteristics and attitude towards MA dispensing

Characteristic	**Agreement with the following Attitude statements**			
	I have the knowledge to counsel women about the process of medication abortion		I feel confident to dispense medication abortion medications	
	**Row n (%)**	**RRR (95% CI)**	**Row n (%)**	**RRR (95% CI)**
**Age**				
<30	94 (63.5)	REF	91 (61.5)	REF
30 – 39	134 (62.9)	0.99 (0.84 – 1.16)	135 (63.4)	1.03 (0.88 – 1.2)
40 – 49	63 (75.9)	**1.20 (1.01 – 1.42)**	64 (77.1)	**1.25 (1.05 – 1.49)**
50 – 59	36 (64.3)	1.01 (0.80 – 1.27)	37 (66.1)	1.07 (0.86 – 1.35)
60+	27 (81.8)	**1.29 (1.05 – 1.58)**	28 (84.9)	**1.38 (1.14 – 1.67)**
**Gender**				
Man	95 (67.4)	Ref	99 (70.2)	Ref
Woman	254 (65.6)	0.98 (0.86 – 1.12)	251 (65.2)	0.93 (0.82 – 1.06)
**Rurality**				
1 (Metropolitan)	244 (64.6)	Ref	245 (64.8)	Ref
2-5 (Regional/Rural)	93 (69.4)	1.08 (0.94 – 1.23)	92 (68.7)	1.05 (0.92 – 1.21)
6-7 (Remote/Very Remote)	17 (85.0)	**1.32 (1.08 – 1.61)**	18 (90.0)	**1.39 (1.18 – 1.63)**
**Year in community pharmacy**				
0-4	78 (65.0)	Ref	74 (61.7)	Ref
5-9	73 (58.4)	0.90 (0.74 – 1.10)	76 (60.8)	0.99 (0.81 – 1.20)
10-14	71 (62.3)	0.96 (0.79 – 1.12)	71 (62.3)	1.01 (0.83 – 1.23)
15-19	35 (83.3)	**1.28 (1.06 – 1.55)**	34 (81.0)	**1.31 (1.07 – 1.61)**
20-24	38 (82.6)	**1.27 (1.05 – 1.53)**	39 (84.8)	**1.37 (1.14 – 1.66)**
25-29	12 (54.6)	0.84 (0.56 – 1.26)	14 (63.6)	1.03 (0.73 – 1.46)
30+	47 (73.4)	1.13 (0.93 – 1.38)	47 (73.4)	1.19 (0.97 – 1.46)
**Community Pharmacy type**				
Independent Pharmacy	143 (66.2)	Ref	142 (65.7)	Ref
Banner Group	179 (64.9)	0.98 (0.86 – 1.14)	182 (65.9)	1.00 (0.88 – 1.14)
Friendly Society	25 (83.3)	**1.26 (1.04 – 1.52)**	23 (76.7)	1.17 (0.94 – 1.45)
Other e.g., GP clinic, not stated	7 (63.6)	0.96 (0.61 – 1.52)	8 (72.7)	1.11 (0.76 – 1.61)
**Dispense MS -2 Step Medication**				
No	208 (55.8)	Ref	211 (56.6)	Ref
Yes	146 (91.3)	**1.64 (1.48 – 1.81)**	144 (90.0)	**1.59 (1.44 – 1.76)**
**Qualifications held**				
BPharm				
No	55 (70.5)	Ref	57 (73.1)	Ref
Yes	299 (65.7)	0.93 (0.79 – 1.09)	298 (65.5)	0.90 (0.77 – 1.04)
MPharm				
No	306 (66.4)	Ref	305 (66.2)	Ref
Yes	48 (66.7)	1.01 (0.84 – 1.20)	50 (69.4)	1.05 (0.89 – 1.24)

Note: Bolded results indicate significant findings at $p < 0.05$.

Supplementary Table 4. Relationship between CP characteristics and MA Knowledge

Characteristic	Knowledge Statement					
	In Australia, medication abortion is registered for use up to 9 weeks (63 days) gestation in all states (True)		Efficacy of medication abortion is similar to that of surgical abortion (True)		Misoprostol is administered before mifepristone (False)	
	**Row n (%)**	**RRR (95% CI)**	**Row n (%)**	**RRR (95% CI)**	**Row n (%)**	**RRR (95% CI)**
**Age**						
<30	71 (47.8)	REF	66 (44.6)	REF	68 (45.9)	REF
30 – 39	107 (50.2)	1.05 (0.84 – 1.30)	109 (51.2)	1.15 (0.92 – 1.43)	85 (39.9)	0.87 (0.68 – 1.10)
40 – 49	35 (42.2)	0.88 (0.65 – 1.20)	38 (45.8)	1.03 (0.76 – 1.38)	34 (41.0)	0.89 (0.65 – 1.22)
50 – 59	30 (53.6)	1.12 (0.83 – 1.50)	37 (66.1)	**1.48 (1.14 – 1.92)**	27 (48.2)	1.05 (0.76 – 1.45)
60+	18 (54.5)	1.14 (0.78 – 1.62)	16 (48.5)	1.09 (0.73 – 1.61)	17 (51.5)	1.12 (0.77 – 1.63)
**Gender**						
Man	70 (49.7)	Ref	71 (50.4)	Ref	59 (41.8)	Ref
Woman	190 (49.3)	0.99 (0.82 – 1.21)	191 (49.6)	0.99 (0.81 – 1.19)	170 (44.2)	1.05 (0.84 – 1.32)
**Rurality**						
1 (Metropolitan)	178 (47.1)	Ref	186 (49.2)	Ref	164 (43.4)	Ref
2-5 (Regional/Rural)	172 (53.7)	1.14 (0.94 – 1.38)	71 (53.0)	1.07 (0.89 – 1.30)	55 (41.0)	0.95 (0.75 – 1.19)
6-7 (Remote/Very Remote)	11 (55.0)	1.17 (0.77 – 1.76)	8 (40.0)	0.81 (0.47 – 1.40)	12 (60.0)	1.38 (0.95 – 2.01)
**Year in community pharmacy**						
0-4	67 (55.8)	Ref	52 (43.3)	Ref	58 (48.3)	Ref
5-9	52 (41.6)	0.75 (0.57 – 0.97)	58 (46.4)	1.07 (0.81 – 1.41)	48 (38.4)	0.79 (0.59 – 1.06)
10-14	59 (51.8)	0.93 (0.73 – 1.18)	56 (49.1)	1.13 (0.86 – 1.50)	51 (44.7)	0.93 (0.70 – 1.22)
15-19	21 (50.0)	0.90 (0.64 – 1.26)	24 (57.1)	1.32 (0.95 – 1.84)	13 (31.0)	0.64 (0.39 – 1.04)
20-24	18 (38.1)	0.70 (0.47 – 1.04)	25 (54.4)	1.25 (0.90 – 1.75)	19 (41.3)	0.85 (0.58 – 1.36)
25-29	9 (40.9)	0.73 (0.43 – 1.24)	11 (50.0)	1.15 (0.72 – 1.84)	12 (55.0)	1.13 (0.74 – 1.73)
30+	35 (54.7)	0.98 (0.74 – 1.29)	40 (62.5)	**1.44 (1.09 – 1.91)**	30 (46.9)	0.97 (0.70 – 1.34)
**Community Pharmacy type**						
Independent Pharmacy	108 (50.0)	Ref	107 (49.5)	Ref	84 (38.9)	Ref
Banner Group	134 (48.6)	0.97 (0.81 – 1.16)	142 (51.5)	1.03 (0.87 – 1.24)	129 (46.7)	1.20 (0.97 – 1.48)
Friendly Society	18 (60.0)	1.20 (0.87 – 1.65)	15 (50.0)	1.01 (0.69 – 1.48)	16 (53.3)	1.37 (0.94 – 1.99)
Other e.g., GP clinic, not stated	1 (9.1)	0.18 (0.03 – 1.19)	2 (18.2)	0.37 (0.10 – 1.30)	2 (18.2)	0.47 (1.32 – 1.66)
**Dispense MS -2 Step Medication**						
No	156 (41.8)	Ref	172 (46.1)	Ref	123 (33.0)	Ref
Yes	105 (65.6)	**1.57 (1.33 – 1.85)**	94 (58.8)	**1.27 (1.07 – 1.51)**	108 (67.5)	**2.05 (1.71 – 2.45)**
**Qualifications held**						
BPharm						
No	34 (43.6)	Ref	34 (43.6)	Ref	30 (38.5)	Ref
Yes	227 (49.9)	1.14 (0.87 – 1.50)	232 (51.0)	1.17 (0.89 – 1.53)	201 (44.2)	1.15 (0.85 – 1.55)
MPharm						
No	226 (49.0)	Ref	237 (51.4)	Ref	204 (44.3)	Ref
Yes	35 (48.6)	0.99 (0.77 – 1.28)	29 (40.3)	0.78 (0.58 – 1.05)	27 (37.5)	0.85 (0.62 – 1.16)

Note: Bolded results indicate significant findings at $p < 0.05$.

Supplementary Figure 1. Benefits of MA

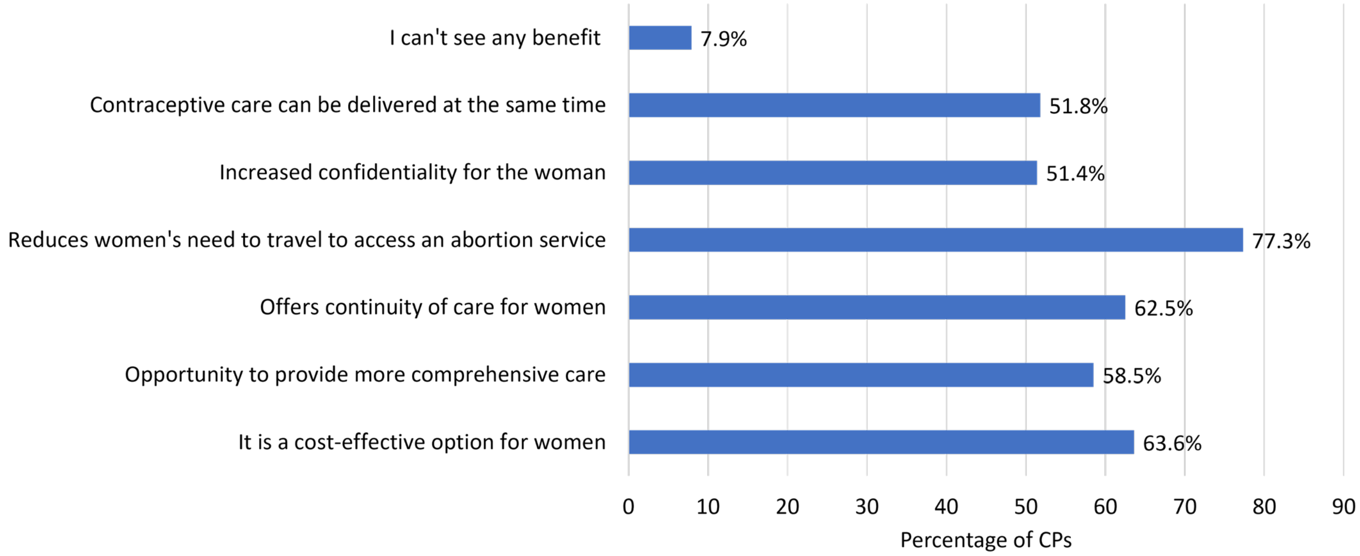

Supplement: Supplementary file 1 — Supplementary file1 (PDF 576 KB) [file 11096_2026_2088_MOESM1_ESM.pdf]
